# Polymorphism of the cytochrome P-450 1A1 (A2455G) in women with breast cancer in Eastern Azerbaijan, Iran

**Published:** 2014-03

**Authors:** Hakimeh Saadatian, Jalal Gharesouran, Vahid Montazeri, Seyyed Abolgasem Mohammadi, Seyyed Mojtaba Mohaddes Ardabili

**Affiliations:** 1 Department of Medical Genetics, Faculty of Medicine, Tabriz University of Medical Sciences, Tabriz, Iran; 2 Department of Thorax Surgery, Faculty of Medicine, Tabriz University of Medical Sciences, Tabriz, Iran; 3 Department of Plant Breeding & Biotechnology, Faculty of Agriculture University of Tabriz, Tabriz, Iran

**Keywords:** Breast cancer, CYP1A1, Polymorphism

## Abstract

***Objective(s):*** Cytochrome P-450 1A1 is an important enzyme in the first phase of the metabolism of some carcinogens such as polycyclic aromatic hydrocarbons (PAHs), as well as estrogen. The present study evaluates the existence of CYP1A1 polymorphism in a number of breast cancer samples.

***Materials and Methods:*** One hundred breast cancer patients and the same number of healthy controls were analyzed for the A2455G polymorphism of cytochrome P-450 1A1 by the polymerase chain reaction-restriction fragment length polymorphism technique.

***Results:*** Frequency of heterozygote genotype (A/G) indicated significant increase in case group (17%) compared to control group (7%) (OR=2.7; 95% CI=1.07-6.89; *P-*value=0.03). The related result of (A/A) genotype showed significantly decreased risk of breast cancer (OR=0.34; *P-*value=0.01). Higher frequency of heterozygotes was mainly observed among premenopausal breast cancer patients.

***Conclusion:*** Our results suggest that the CYP1A1 polymorphism may be useful for predicting breast cancer risk in our study population.

## Introduction

Breast cancer is the leading cause of cancer death among women in the world (1). The majority of breast cancer patients do not have direct family history of breast cancer and they are regarded as sporadic cases (2). It can be concluded that the environment may play a significant role in the development of this type of cancer. 

Cytochrome P-450 (CYP) 1A1 is a xenobiotic metabolizing enzyme (XMEs) which participates in the metabolism of a vast number of xenobiotic components as well as a small number of endogenous substrates (3). 

CYP1A1 encodes aryl hydrocarbon hydrolase (AHH), an enzyme involved in the production of reactive epoxide intermediates from polycyclic aromatic hydrocarbons, steroid hormones, and other aromatic compounds (4). Here, we report the results obtained from the study of potential association between the above mentioned gene variations and susceptibility to breast carcinoma in the population of Eastern Azerbaijan, Iran. 

## Materials and Methods

***Subjects***

This study included 200 unrelated subjects (100 controls and 100 patients), living in Eastern Azerbaijan, Iran. The patient samples were collected from women who were referred to Tabriz Hospitals (Tabriz, Iran) with unilathral primary breast carcinoma, without any radiotherapy background and family history of breast cancer. The patients received modified radical mastectomy or breast conserving surgery. To avoid confounding of ethnicity, we excluded patients from neighboring countries, judged by name, language and place of birth. The patient group had a mean age of 42±9 years. A detailed description of clinical and pathological characteristics of samples has been summarized in Table 1. Control subjects having a mean age of 49±15 years, were comprised of women referred to Hospital Laboratories of Tabriz, Tabriz, Iran showing no evidence of any personal or family history of cancer. The written informed consent was obtained from all subjects.

**Table 1:** Clinical features of breast carcinoma patients

Clinical tumor size	
T1-T2(64)^a^	88.9%
T3(8)a	11.1%
Lymph node status	
N(+)(72) a	72.7%
N(-)(27) a	27.2%
SBR grading	
1 – 2(80) a	86.3%
3(8) a	13.6%

a number of patient that their related pathologic status were available

***DNA extraction and polymerase chain reaction (PCR)***

About 3 ml of peripheral blood sample was collected from each individual participated in the study and used for DNA isolation by salting out procedure. The samples were centrifuged and the pellet was resuspended in RBC lysis buffer. Then, the pellet was mixed with WBC lysis buffer (10 mM Tris-HCl [pH 8.0], 0.5 mM EDTA, 1M NaCl) in the presence of 200 µl of 10% SDS and 50 ul proteinase K (20 mg/ml) and incubated at 37°C for 16 hr following to addition of saturated salt solution. DNA was precipitated by 1-3 ml of isopropanol and washed using 1 ml of 70% ethanol. The DNA pellet was dissolved in sterile distilled water.

 The PCR reaction was prepared using 100 ng of template DNA, 0.03 µg of each of forward (5´CTGTCTAGGCTGGTTCTCCACAAGC3´) and reverse (5´GGC TATCCTGCTGCAACGGGTGGAA3´) primers, 1.25 units of Taq DNA polymerase (Cinnagen), 200 µM of dNTPs, 2.5 µl of 10x PCR buffer (50 mM KCl, 10 mM Tris-HCl, pH 8.3) and 2 mM MgCl_2_. PCR primers were designed as described by Cascorbi *et al* (5). The volume was adjusted to 25 µl by distilled H_2_O. The cycling conditions were as follows: after an initial denaturation at 94°C for 5 min, 31 cycles of polymerization were carried out by denaturation at 94°C for 30 sec, hybridization at 62.5 °C for 30 sec, and extension at 72°C for 30 sec. The final extension was performed at 72°C for 7 min. The PCR reaction yielded a DNA fragment of 204 bp length.

***Genotyping***

 A2455G mutation in CYP1A1 gene was detected by PCR-RFLP approach as described by Cascorbi *et al* (5) with minor modifications. The 204 bp fragment was digested by BsrDI restriction enzyme. The enzyme recognizes 5’ GCAATGNN 3’ restriction site. A BsrDI restriction site is omitted when the A/G mutation occurs. For every RFLP reaction, 10 µl of PCR product, 1 unit of BsrDI restriction enzyme with 2 µl of related buffer were used. The volume of the reaction was adjusted to 30 µl by distilled water. The reaction mixture was incubated at 55°C for 1-16 hr and the resulting fragments were fractionated on a 2% agarose gel.

**Table2:** Association between CYP1A1 polymorphisms and breast cancer

Polymorphism	Cases	Controls	Odds ratio
A/A	82(82)^a^	93(93)	0.34 (*P-*value=0.01)
A/G	17(17)	7(7)	2.70 (*P-*value=0.03)
G/G	1(1)	0(0)	-
A/G + G/G	18(18)	7(7)	2.92(*P-*value=0.019)
Allele frequency	*P*=0.9	*P*=0.96	
	q=0.1	q=0.04	

a Numbers in parentheses are percentages

***Statistical analysis***

The results were analyzed by SPSS version 16. The genotype frequencies of the gene were tested for Hardy-Weinberg equilibrium for both patient and control groups using the χ^2^ test. The same test was used to evaluate the association between the disease and different genotypes. For the estimation of breast cancer risk, we used a logistic regression model to calculate the odds ratio and 95% confidence interval (CI) for each genotype. The clinicopathologic parameters studied here were: age, nodal status, SBR (Scraff, Bloom and Richardson) tumor grade and clinical tumor size.

## Results

**Figure.1:**
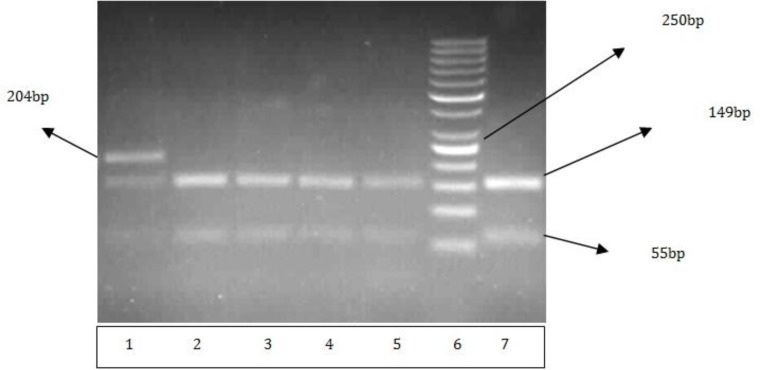
Polymerase chain reaction-restriction fragment length polymorphism agarose gel electrophoresis of cytochrome1A1 (A2455G). Lane 1 referred to heterozygote genotype and the rest of the samples, except for lane 6 that is 50 bp marker, are indicative of wild homozygote

In the present study allele frequencies of the variant allele CYP1A1 (A2455G) among the cases and controls were 0.1 and 0.04, respectively. The genotype frequencies and the results of the case control study have been summarized in Table 2. The wild and variant alleles have been designated as A and G respectively. As it can be concluded from Table 2, the risk of breast cancer was increased among heterozygote women carrying the variant allele G (462Val) (odds ratio 2.70, 95% CI 1.075-6.89, *P-*value=0.03. The risk appeared to be increased predominantly in pre-menopausal subgroup (odds ratio 11.66, 95% CI 1.5 -90.76, *P-*value=0.004. The related result of genotype assessment showed significantly decreased risk of breast cancer for homozyghote wild genotype (A/A) (OR=0.34; *P-*value=0.01). Genotype distribution in both case and control groups did not diverge significantly from Hardy-Weinberg equilibrium. In addition, we found no significant discrepancy among (A/G) genotype and clinic-pathological characteristics regarding the tumor size and grade. According to lymph node status, the frequency of heterozygote genotype was slightly increased in positive lymph nodes. Figure 1 shows the PCR-RFLP agarose gel electrophoresis of CYP1A1 (A2455G) gene.

## Discussion

A number of cancers have been shown to be associated with CYP1A1 genotypes (6). In the present study, we have shown that heterozygote individuals for allele 2455G (462Val) have a significantly higher risk of breast cancer compared to the other genotypes and wild homozygotes showed statistically significant decreased risk. Surekha *et al *reported the association of A2455G variant with increased risk of breast cancer in women with premenopausal status in Indian population (6), while the reports for the other ethnicities were different. In Caucasian populations, Theodoros *et al* found that homozygous carriers of A2455G (G/G) exhibit higher risk of breast cancer compared to the heterozygous carriers and homozygous normal individuals (7). In a meta-analysis study, Chen *et al* revealed that G/G genotype is associated with a trend of reduced breast cancer risk, both in East-Asian women and in pre-menopausal women worldwide (8). This finding differs from the results reported by Japanese and Chinese indicating significantly reduced risk of breast cancer in women with the heterozygote (A/G) genotype instead of homozygous carriers (G/G)(9, 10).

The higher frequency of heterozygotes for Val substitution among pre-menopausal breast cancer patients in our study, suggests the role of this variation in early onset of the disease. Similar results have been reported by other investigators (6, 11). However, increased risk of breast cancer has been reported for women carrying the Val variant in postmenopausal status which can be explained by prolonged exposure of affected individuals to environmental carcinogens 12).

It has to be noticed that about 85.9% of the patients participated in the present study were in premenopausal status and since the rate of heterozygotes within this group was greater than those of post menopause patients, this may indicate an increased occurrence of breast cancer at younger ages in our population and hence CYP1A1 genotype may have a role in early onset of breast cancer. 

In our study, no statistically significant association between the genetic polymorphism and clinico-pathological characteristics of breast cancer was observed. The frequency of 2455G (Val) allele was found to be slightly increased in breast cancer patients with node-positive status which disagrees with a similar study done in India (7).

Studies explain the importance of gene-environment interaction in carcinogenesis (12, 13). Smoking is a major way of PAH exposure (14). Also, PAHs (and heterocyclic amines) are formed when meat is cooked at high temperatures (15). Regarding these findings, the important issues are examining relationship between CYP1A1 polymorphism and breast cancer risk considering environmental factor (especially dietary factor) that has not been evaluated in our study. Further studies using large sample size is also necessary to confirm the results obtained in the present study. 

## Conclusion

The result obtained from the present study indicates that the carriers of 462Val have a significantly higher risk of breast cancer than those are homozygous normal which was outstanding in premenopausal subgroup. Normal homozygote genotypes showed decreased risk of breast cancer. Affected individuals carrying 426Val variant allele do not have distinct clinico-pathological characteristics. 
